# Heat Treatment-Induced Microstructure and Property Evolution of Mg/Al Intermetallic Compound Coatings Prepared by Al Electrodeposition on Mg Alloy from Molten Salt Electrolytes

**DOI:** 10.3390/ma14061407

**Published:** 2021-03-14

**Authors:** Tianyu Yao, Kui Wang, Haiyan Yang, Haiyan Jiang, Jie Wei, Weiping Wu, Hezhou Liu, Qudong Wang, Wenjiang Ding

**Affiliations:** 1 National Engineering Research Center of Light Alloy Net Forming, Shanghai Jiao Tong University, 800 Dongchuan Road, Minhang District, Shanghai 200240, China; yty123261002@sjtu.edu.cn (T.Y.); jianghy@sjtu.edu.cn (H.J.); weijiesjtu@sjtu.edu.cn (J.W.); wangqudong@sjtu.edu.cn (Q.W.); wjding@sjtu.edu.cn (W.D.); 2 The State Key Lab of Metal Matrix Composites, Shanghai Jiao Tong University, 800 Dongchuan Road, Minhang District, Shanghai 200240, China; hzhliu@sjtu.edu.cn; 3 Laboratory of Thin Film Optics, Shanghai Institute of Optics and Fine Mechanics, Chinese Academy of Sciences, 390 Qinghe Road, Jiading District, Shanghai 201800, China; wuwp@siom.ac.cn

**Keywords:** intermetallic compound, Zn layer, Al coating, heat treatment, Mg_17_Al_12_, Mg_2_Al_3_

## Abstract

A method of forming an Mg/Al intermetallic compound coating enriched with Mg_17_Al_12_ and Mg_2_Al_3_ was developed by heat treatment of electrodeposition Al coatings on Mg alloy at 350 °C. The composition of the Mg/Al intermetallic compounds could be tuned by changing the thickness of the Zn immersion layer. The morphology and composition of the Mg/Al intermetallic compound coatings were characterized using scanning electron microscopy (SEM), X-ray diffraction (XRD), and electron backscattered diffraction (EBSD). Nanomechanical properties were investigated via nano-hardness (nHV) and the elastic modulus (EIT), and the corrosion behavior was studied through hydrogen evolution and potentiodynamic (PD) polarization. The compact and uniform Al coating was electrodeposited on the Zn-immersed AZ91D substrate. After heat treatment, Mg_2_Al_3_ and Mg_17_Al_12_ phases formed, and as the thickness of the Zn layer increased from 0.2 to 1.8 μm, the ratio of Mg_2_Al_3_ and Mg_17_Al_12_ varied from 1:1 to 4:1. The nano-hardness increased to 2.4 ± 0.5 GPa and further improved to 3.5 ± 0.1 GPa. The Mg/Al intermetallic compound coating exhibited excellent corrosion resistance and had a prominent effect on the protection of the Mg alloy matrix. The control over the ratio of intermetallic compounds by varying the thickness of the Zn immersion layer can be an effective approach to achieve the optimal comprehensive performance. As the Zn immersion time was 4 min, the obtained intermetallic compounds had relatively excellent comprehensive properties.

## 1. Introduction

Mg and its alloys have become increasingly promising candidates for structural materials in industrial applications in recent years, due to their low density and high specific strength. However, their poor surface properties, such as corrosion and wear resistance, cannot fulfill industrial demands, thus hindering wider application [1,2,3]. Inspired by the design of new alloys, surface modification has been regarded as a practical and effective approach to improve surface properties. Various methods of surface modifications, including physical vapor deposition (PVD), chemical vapor deposition (CVD), chemical conversion, anodizing, electrodeposition, electro-less plating, organic coating, laser and ion beams, have been adopted in essentially all cases [4,5,6,7,8,9,10].

Among them, surface alloying of Mg alloys with Al has been attracting more and more attention since the formation of Mg/Al intermetallic compounds can improve corrosion and wear resistance. The typical intermetallic compounds formed by Mg and Al, that is, Mg_17_Al_12_ and Mg_2_Al_3_, show desirable corrosion resistance and wear resistance. Many studies [11,12] have also demonstrated that Mg/Al intermetallic compounds, especially Mg_17_Al_12_, exhibited excellent corrosion resistance in chloride solutions. As the Mg matrix is coated by Mg_17_Al_12_ in chloride solutions, the Mg_17_Al_12_ maintains chemical stability in a wide pH range, thus improving the corrosion resistance of the alloy.

Numerous studies have been focused on achieving a surface layer of Mg/Al intermetallic compounds on Mg and its alloys, which are composed of Mg_17_Al_12_ and Mg_2_Al_3_. Bu et al. [13] synthesized Mg/Al intermetallic compounds by the cold spray process onto AZ91D magnesium (Mg) substrates. Zhao et al. [14] achieved an Mg/Al intermetallic compound coating by Mg/Al twin-wire arc spraying. Zhu et al. [11] prepared an Mg/Al-alloyed coating onto the surface of magnesium alloy AZ91D via a powder metallurgy process. Although Mg/Al intermetallic compounds can be successfully produced using these methods, they could also pose some problems. Firstly, the preparation process requires a high working temperature, and thus makes the thickness and the uniformity of the intermetallic compounds layer uncontrollable. Secondly, the uncontrollable diversity of the thickness and the uniformity of the intermetallic compounds layer leads to poor corrosion resistance, so the layer may not be capable of protecting the matrix sufficiently. What is more, the composition of the Mg/Al intermetallic compounds layer is determined and unmodifiable, which makes it impossible to improve the performance by tuning different components.

It is well-documented that electrodeposition is one of the most effective methods to fabricate coatings and films because of its convenience, simplicity, and cost-effectiveness [15]. Furthermore, the compactness and thickness of the coatings can be controlled by altering the processing parameters of electrodeposition. Electrodeposition is the optimal solution to get Al coatings onto Mg alloy with regular structure, smooth surface, and controllable thickness [16]. After electrodeposition, heat treatment is employed to form Mg/Al intermetallic compounds [17]. The thickness and quality of the Al coatings can be tailored through optimization of the processing parameters of electrodeposition. On the other hand, it has been demonstrated [18,19] that the addition of Zn favors the formation of Mg/Al intermetallic compounds through diffusion at a low temperature. Therefore, if the process of Zn immersion is performed before the electrodeposition of Al onto Mg alloy, it is expected that the introduction of Zn could facilitate the formation of Mg_17_Al_12_ and Mg_2_Al_3_ phases during the heat treatment [20], which helps to produce the composition-controllable Mg/Al intermetallic compound coatings.

In the present study, a novel two-step method of forming an Mg/Al intermetallic coating that is enriched with Mg_17_Al_12_ and Mg_2_Al_3_ was developed. The composition of Mg_17_Al_12_ and Mg_2_Al_3_ could be tuned by changing the thickness of the Zn immersion layer so that Mg/Al intermetallic coatings with different performance preferences could be obtained. Step one was to prepare the Mg–Zn–Al composite coatings through the electrodeposition of Al from AlCl_3_–NaCl–KCl (80–10−10 wt.%) molten salt electrolytes onto AZ91D Mg alloy. Step two was to obtain the layer of thick Mg/Al intermetallic coatings, which were enriched with Mg_17_Al_12_ and Mg_2_Al_3_, through a vacuum heat treatment at 350 °C. The effect of heat treatment on the morphology and microstructures of Al was studied systematically. The morphology and composition of the Mg/Al intermetallic coatings were characterized using scanning electron microscopy (SEM), X-ray diffraction (XRD), and electron backscattered diffraction (EBSD). Additionally, the corrosion performance was also evaluated and the corrosion behavior was studied through potentiodynamic (PD) polarization.

## 2. Materials and Methods

### 2.1. Zn Immersion

AZ91D Mg alloy (as-cast, Mg–9 wt.% Al–1 wt.% Zn) was used in this research. Specimens were cut into small bars with a size of 5 mm × 5 mm × 100 mm. Before Zn immersion, each sample was ground with SiC papers to 1500 grit, and then degreased ultrasonically in an ethanol solution for 3 min. A layer of Zn was obtained by the following steps: pickling → conditioning → activation → Zn immersion. The solution compositions and operation conditions are shown in Table 1. Different Zn immersion times, 2 min, 4 min, 6 min, and 8 min, were adopted in this experiment.

### 2.2. Electrodeposition and Heat Treatment

Sodium chloride (NaCl, AR, 99.9% purity) and potassium chloride (KCl, AR, 99.9% purity) were kept in a vacuum drying chamber at 300 °C for 72 h before the experiment to remove moisture. Anhydrous Al chloride (AlCl_3_, AR, 99.8% purity) was used as received. AlCl_3_, NaCl, and KCl (80–10−10 wt.%) were mixed and melted at 150 °C in a radius flask on a temperature-controlled heater inside a glove box (Mikrouna Upure 1220/750/900, Shanghai, China), which was purged with high-purity Ar gas. The mixture was continuously stirred with a magnetic stirrer for more than 4 h to yield a homogeneous light yellow liquid.

All of the electrodeposition experiments were carried out using an electrochemical workstation (CHI655D, Shanghai Chenhua Device Company, Shanghai, China) in the glove box. An Al plate (99.99%, 350 mm^2^) and a pure Al bar (99.99%, diameter Φ = 0.5 mm) were used as the counter electrode and the reference electrode. The Mg alloy bar after the Zn immersion pretreatment was connected as the cathode. A 10 mm-wide interval was maintained between the cathode and the anode. All of the electrode materials were cleaned and dried before they were transferred into the glove box. Cathodic deposition of Al was performed under a constant current mode at 150 °C with a current density of 50 mA·cm^−2^, and the process of electrodeposition lasted for 10 min.

After the electrodeposition, the samples were cleaned completely using anhydrous acetone in an ultrasonic bath for 2 min, rinsed with deionized water, and then dried in the vacuum drying chamber. The samples of the electrodeposition Al coatings on the Mg alloys were heat-treated in a vacuum oven (SX2-4-10Z, Shanghai Boxun Device Company, Shanghai, China) with a vacuum pressure of <10^−3^ Pa at 350 °C for 4 h and cooled down to room temperature in the furnace.

### 2.3. Characterization

A field emission scanning electron microscope (FE-SEM, SIRION200, FEI, Hillsboro, OR, USA) was used to observe the surface and cross-sectional morphologies of the Al coatings. The samples were examined by X-ray diffraction (XRD, D/MAX2000 V, Rigaku, Japan) to identify the constituents and the crystal structure by the 2θ/θ scanning mode. The data was collected in a 2θ = 30°–80° range with a scanning speed of 5°·min^−1^ with a 0.01 degree (2θ) step size. The obtained data were compared with the International Centre for Diffraction Data (ICDD) Card No. 004-0787 to identify the element.

Nanoindentation tests were performed on the cross-section of each sample with a nano-hardness instrument (NST, CSM0200, CSM, Lausanne, Switzerland). (The nanoindentation tests were conducted on the surface of the Zn layers, since they were too thin.) The indentation load was set at 2000 μN and the loading/unloading rate was kept constant at 4000 μN per minute. The stabilization time between the loading and unloading stages was fixed at 5 s. There were 5 indentations taken, and the distance between each indentation was 10 μm to eliminate the overlap of the indentations. The nano-hardness was calculated as the average of the 5 data points. The elastic modulus (EIT) and nano-hardness (nHV) were calculated from the load-displacement curves as per [21]. The values of the EIT were determined from the slope (S) of the unloading curve by the equation below. Specifically, it was a measurement of the elastic properties of the different surfaces [22].

(1) $$E_{r}=\frac{S}{2}\sqrt{\frac{\pi }{A}}$$

where *A* is the projected area after the indentation.

The hydrogen evolution experiments were performed to measure the cumulative corrosion evolved during the extended period of immersion in 3.5 wt.% NaCl solution at room temperature for 136 h. The NaCl solutions were prepared with deionized water and were not deaerated. An upside-down burette tube filled with the same solution was placed over the sample during the tests to collect evolved hydrogen gas. Before the immersion, the samples were cold-mounted in epoxy resin with a copper wire attached, and an exposed surface area of 0.25 cm^2^ (5 mm × 5 mm) was adopted to assure that all the corrosion occurred here. For each kind of sample, the hydrogen evolution experiments were carried out three times. In order to evaluate the corrosion resistance, the electrochemical behavior was also studied by potentiodynamic polarization (PD). The PD tests were performed in the 3.5 wt.% NaCl solution at room temperature. The counter electrode and the reference electrode were a graphite electrode and a saturated calomel electrode (SCE), respectively. All the samples remained at the open circuit for 30 min to reach a steady value before the PD tests. Scans were obtained from 100 mV below the open circuit potential (OCP) and scanned upwards at a rate of 0.5 mV/s. The corrosion current density (i_corr_) and corrosion potential (E_corr_) were calculated by the Tafel extrapolation.

## 3. Results and Discussion

### 3.1. As-Deposited Microstructures

**Figure 1:**
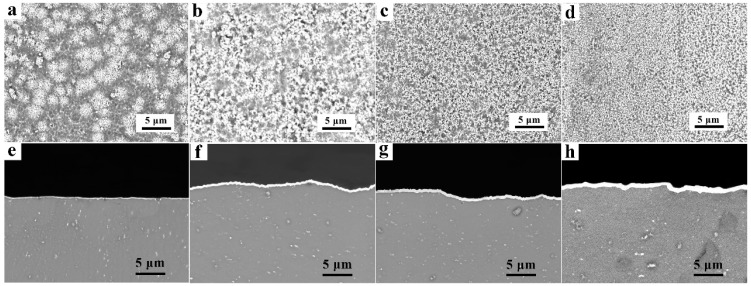
(**a**–**d**) The SEM planar view and (**e**–**h**) the cross-sectional morphologies of the Zn layers on the AZ91D Mg alloys with Zn immersion for (**a**,**e**) 2 min, (**b**,**f**) 4 min, (**c**,**g**) 6 min, and (**d**,**h**) 8 min.

Figure 1a–d shows the planar view SEM images of the Zn layers on the AZ91D alloys that underwent different immersion times. Figure 1e–h depicts the cross-sectional morphologies of the Zn layer on the AZ91D Mg alloys. It can be seen that the surface and cross-sectional morphologies of the Zn layer varied significantly. As the Zn immersion time reached 2 min, shown in Figure 1a,e, the distribution of the Zn particles on the Mg alloys was not uniform, which means an inhomogeneous and discontinuous Zn layer was obtained. The cross-sectional morphology showed that the Zn layer was quite thin, with a thickness of 0.2 μm. With the increase of the Zn immersion time, the densification and thickness of the Zn layer increased gradually and obviously. After an 8-min Zn immersion, as shown in Figure 1d,h, the surface of the Mg alloy was completely covered by Zn, and a uniform and dense Zn layer with good flatness was obtained. The matrix was dark grey, while the Zn layer became bright white, and the thickness was increased to 1.8 μm since there was abundant Zn deposited on the Mg alloy. Controlling the time of the Zn immersion is a simple and effective method to adjust the compactness and thickness of the Zn layer.

**Figure 2:**
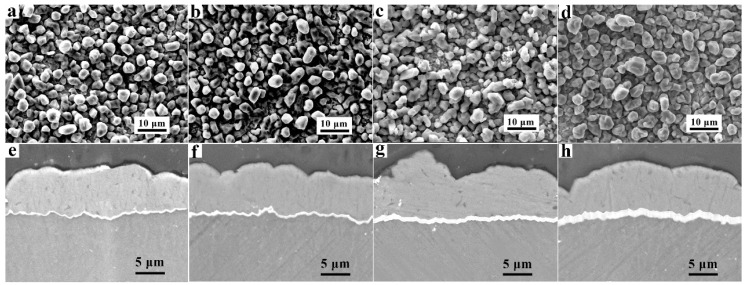
(**a**–**d**) The SEM planar view and (**e**–**h**) the cross-sectional morphologies of the Al coatings on the AZ91D Mg alloy with Zn immersion for (**a**,**e**) 2 min, (**b**,**f**) 4 min, (**c**,**g**) 6 min, and (**d**,**h**) 8 min.

Figure 2a–d shows the planar view SEM images of the Al coating on the Zn-immersed AZ91D alloys with different immersion times. The surface of the Al coating was compact and uniform, which had no significant change with increases in the Zn immersion times. Such a finely deposited Al coating was mainly attributed to the Zn immersed on the substrate, compared with the Al coating on bare AZ91D, according to our previous work [23]. Figure 2e–h demonstrates the cross-sectional morphologies of the Zn-immersed AZ91D Mg alloys with an Al coating. A general morphology of the sandwich structure can be observed: a grey Al layer coated on Mg substrate, sandwiched with a Zn layer.

Since the same amount of electric charge was applied during the process of Al electrodeposition, the thickness of the electrodeposition Al coatings obtained should be consistent. As shown in Figure 2e–h, the thickness of the electrodeposition Al coating obtained from the electrolyte with different Zn immersion times was almost identical, and the average thickness of the Al electrodeposits obtained was 7 ± 3 μm. It is shown that with increasing Zn immersion times, the thickness of the Zn layers reasonably increased. The thickness of the Zn layers varied from 0.2 μm to 1.8 μm, which is in accordance with the results in Figure 1. In addition, there were no gaps between the Al coatings and the Zn layer, or between the Zn layer and the substrate. It is indicated that the electrodeposited Al coating, Zn layer, and the Mg alloy substrate exhibited good interfacial bonding.

**Figure 3:**
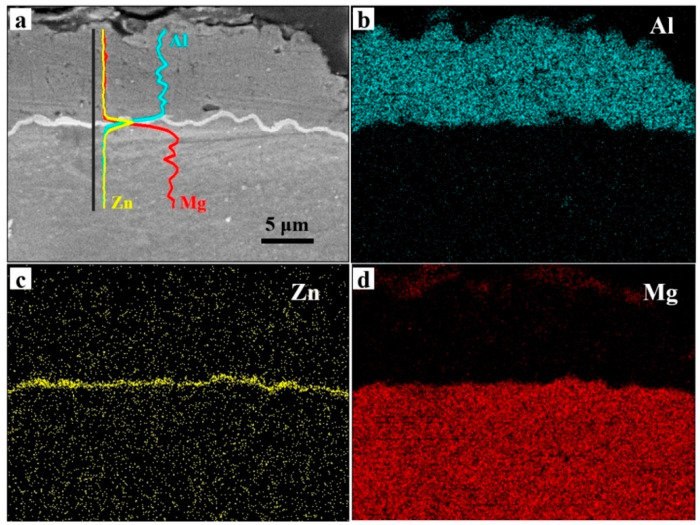
The Energy Dispersive X-Ray Spectroscopy (EDXS) analysis of the electrodeposition Al coatings obtained on the AZ91D Mg alloy with Zn immersion, (**a**) the cross-sectional morphology, (**b**–**d**) the EDXS analysis.

To further confirm the composition of the sandwich structure, Energy Dispersive X-Ray Spectroscopy (EDXS) analysis was carried out on the interfaces of the Al coating on the AZ91D Mg alloy with Zn immersion, and the typical results are shown in Figure 3. The line profiles of these elements were superimposed onto the SEM micrograph. The EDXS mappings show that Al, Zn, and Mg were distributed throughout the top, middle, and bottom layers of the sandwich structure, respectively.

**Figure 4:**
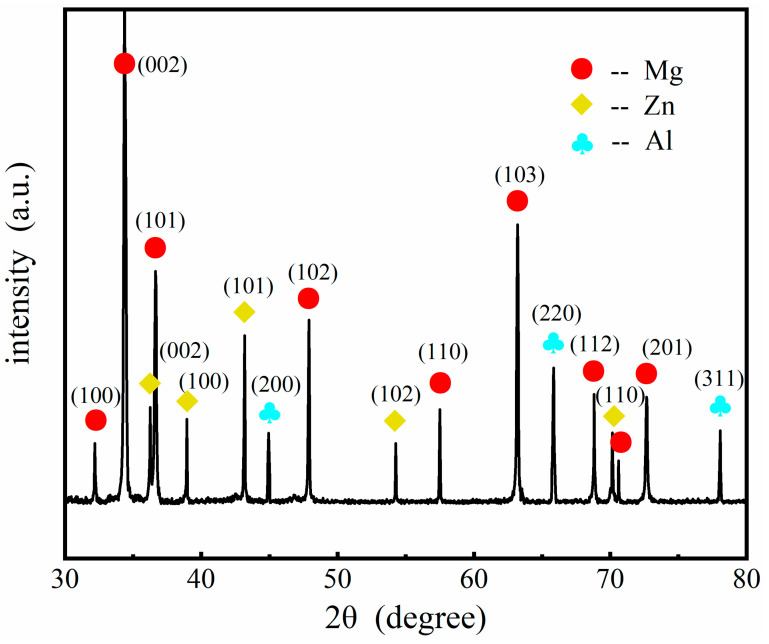
The typical XRD patterns of the electrodeposition Al coatings obtained on the AZ91D Mg alloy.

To identify the composition and phase structure, XRD analysis was carried out on the Al coating of the samples, and the typical XRD patterns of the electrodeposition Al coatings are shown in Figure 4. The strong diffraction peaks could be indexed to the Al coatings, Zn layer, and the Mg alloy substrate, respectively. The peaks of the Al coatings are associated with (200), (220), and (311) planes of its face-centered cubic structure, confirming the formation of a metallic and pure Al. The diffraction signals from the Zn layer and the substrate were also detected, due to the thinness of the Al coating and Zn dipping film. The peaks of Mg had the strongest intensity because the Zn layer and Al coatings were not thick enough.

### 3.2. Microstructures after Heat Treatment

**Figure 5:**
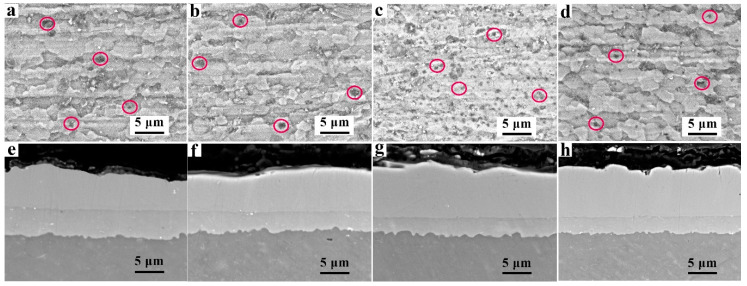
The SEM planar view and (**e**–**h**) the cross-sectional morphologies of the Al coating after 350 °C heat treatment for 4 h with Zn immersion for (**a**,**e**) 2 min, (**b**,**f**) 4 min, (**c**,**g**) 6 min, and (**d**,**h**) 8 min.

Figure 5a–d shows the planar view SEM images of the Al coating on the Zn-immersed AZ91D alloys after heat treatment of 350 °C for 4 h. The surface morphology of the Al coating transformed from particle-like (Figure 2) to a flat shape after heat treatment. Some tiny pinholes, formed during the heat treatment, were observed and circled in the planar view SEM images. The outer layer of the coatings was porous and thus, the inner layer could participate in corrosion processes, affecting the performance of corrosion resistance. The surface morphologies of the Al coating remained unchanged with the increase of the Zn immersion time. Figure 5e–h demonstrates the cross-sectional morphologies of the Zn-immersed AZ91D Mg alloys with Al coating after heat treatment. The major change in appearance was the variation of the Zn layer from a thin white to a thick grey one. Clearly, the inter-diffusion of the elements indeed occurred across the interface, indicating that the metallurgic bonding might be formed during the heat treatment process [24].

**Figure 6:**
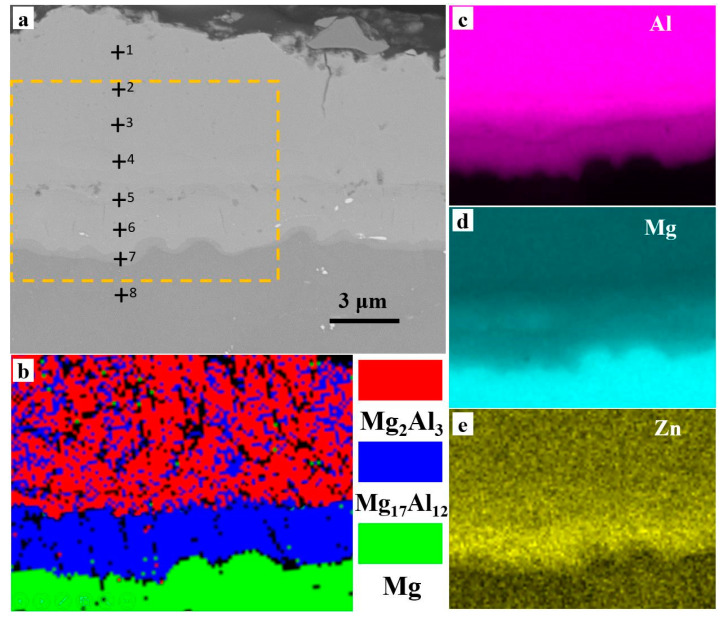
The Electron Backscatter Diffraction (EBSD) and the EDXS analysis of the Al coating after a 350 °C heat treatment for 4 h. (**a**) the cross-sectional morphology, (**b**) EBSD analysis, (**c**–**e**) the EDXS analysis.

The observed variation of the Al coating and Zn layer might be ascribed to the phase transformation near the interface, which was generally induced by the heat treatment [12]. To understand in-depth the crystallinity evolution, the heat-treated samples with Zn immersion for 8 min were examined. Figure 6a demonstrates the overall cross-sectional morphologies of the sample, in which the EDXS areas and the Electron Backscatter Diffraction (EBSD) region were marked. The EDXS mappings of the selected region in Figure 6a are shown in Figure 6c–e, and the EDXS spot analysis of the samples is shown in Table 2. As shown, the Mg/Al atomic ratios of the two layers were approximately 41:59 and 57:40, respectively. As a result, the phases could be identified as γ-phase (Mg_2_Al_3_) in the surface layer, and β-phase (Mg_17_Al_12_) in the middle layer, respectively. Correspondingly, the EBSD inverse pole figure (IPF) map shown in Figure 6b also indicates that the surface was composed of Mg_2_Al_3_, and the middle layer was composed of Mg_17_Al_12_.

**Figure 7:**
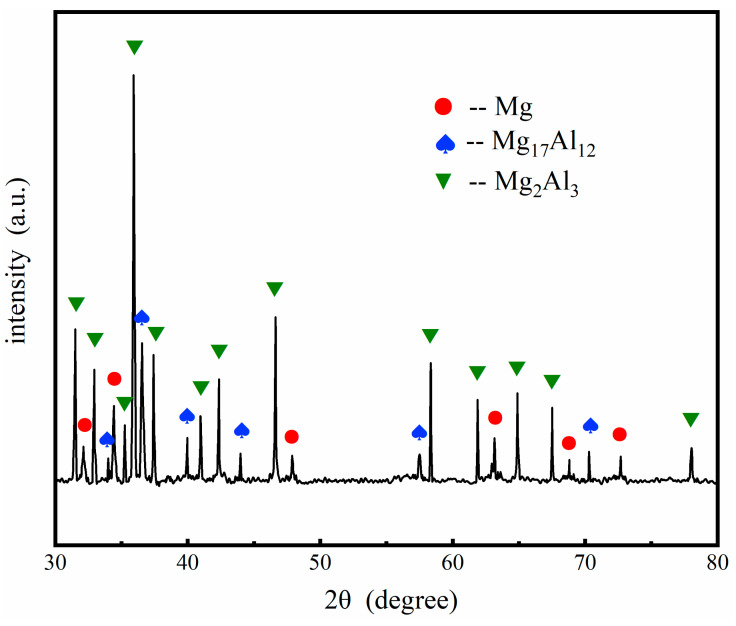
The typical XRD patterns of the Al coating after 350 °C heat treatment for 4 h.

The composition of the samples was further verified by the XRD pattern, as presented in Figure 7. Apart from the Mg alloy substrate, γ-phase and β-phase were also identified in the XRD pattern. Consequently, the double-layer coatings consisted of β-phase (Mg_17_Al_12_) and γ-phase (Mg_2_Al_3_).

After heat treatment, Zn could be detected in the energy spectrum (shown in Figure 6e), but no intermetallic compounds containing Zn were formed. The intermetallic compounds formed with the Zn layer after heat treatment had the same composition and structure as those formed without Zn addition. It indicated that, as a transition layer, Zn could not change the formation of β-phase (Mg_17_Al_12_) and γ-phase (Mg_2_Al_3_). A probable reason is that Zn functioned as a solute and diffused into aluminum.

**Figure 8:**
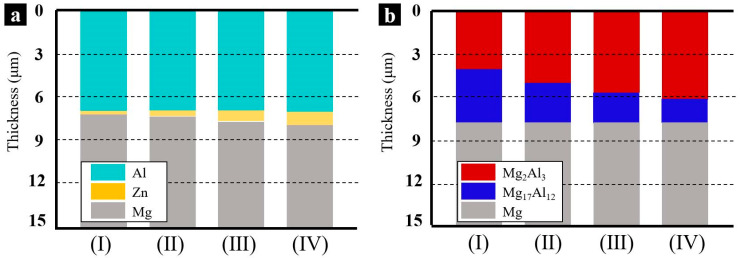
The thickness variations of the intermetallic compounds β-phase (Mg_17_Al_12_) and γ-phase (Mg_2_Al_3_) with various Zn immersion times (I) 2 min, (II) 4 min, (III) 6 min, and (IV) 8 min. (**a**) electrodeposition Al coatings obtained on the AZ91D Mg alloy, (**b**) the Al coatings after a 350 °C heat treatment for 4 h.

It has been confirmed that the Mg/Al intermetallic compound coatings enriched with β-phase (Mg_17_Al_12_) and γ-phase (Mg_2_Al_3_) had already been prepared. According to the measurements in Figure 5, the thickness variations of the intermetallic compounds β-phase (Mg_17_Al_12_) and γ-phase (Mg_2_Al_3_) with various Zn immersion times are shown in Figure 8. The thicknesses of the double-layer coating of the samples with Zn immersion for 2 min, 4 min, 6 min, and 8 min were almost the same, approximately 7 ± 1 μm. However, there was a significant difference in the composition of the two compounds. When the Zn immersion time was 2 min, the thickness of the obtained Zn layer was 0.2 μm, and the ratio of the γ-phase and β-phase, determined by the thickness in the cross-sectional morphologies, was approximately 1:1. With the increase of the thickness of the Zn layer, the proportion of the γ-phase increased, while that of β-phase decreased. When the thickness of the Zn layer reached 1.8 μm, the ratio of the γ-phase and β-phase was approximately 4:1.

The variations of the thickness of the Zn layer made the main contribution to the different ratios of the two Mg/Al intermetallic compounds. The Zn layer played a predominant role in inhibiting the inter-diffusion between the Mg and Al. As the Zn layer was as thin as 0.4 μm, it appeared porous and non-uniform (as shown in Figure 1). The inter-diffusion, especially from the Mg substrate to Al, was abundant and sufficient, so the β-phase, which was enriched with Mg, was preferentially formed. When the thickness of the Zn layer was 1.8 μm, it became thick and dense, which made the diffusion process more difficult. After heat treatment, the Mg content decreased. As a result, only a little β-phase was formed and the majority of the Mg/Al intermetallic compounds were γ-phase.

### 3.3. Nanomechanical Coating Properties

**Figure 9:**
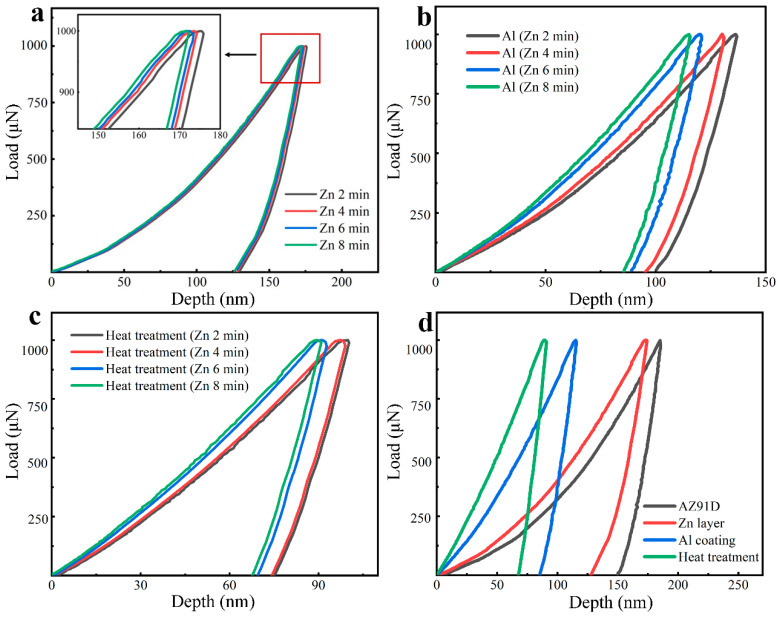
Load–displacement curves obtained from the nanoindentation tests on (**a**) Zn layers (insert is the larger drawing of the rectangular framed region), (**b**) Al coatings, (**c**) Al coatings after heat treatment, and (**d**) the comparison of the load–displacement curves of the AZ91D substrate, the Zn-immersed, the Al-coated, and the heat-treated samples.

The nanomechanical behaviors of the Zn layers and Al coatings before and after heat treatment were evaluated by nanoindentation tests. The load–displacement curves of the samples obtained from the nanoindentation tests are shown in Figure 9. For comparison, the mechanical properties of the AZ91D Mg substrate were also tested. The nano-hardness and the elastic modulus determined by the load–displacement are listed in Table 3.

Figure 9a shows the load–displacement curves of the AZ91D substrate immersed in Zn with different times. There was a little discrepancy among the curves. This was attributed to the increased thickness of the Zn layer, which can resist more deformation than the AZ91D substrate under loading [25]. Figure 9b depicts that the nano-hardness of the Al coatings obtained via electrodeposition increased obviously with the increase of the thickness of Zn layers. This means that the nano-hardness of the Al coatings was highly related to the thickness of the Zn layer. Notice that in Figure 9c, the nano-hardness of the intermetallic compound layers formed after heat treatment also increased significantly with the increase of the thickness of the Zn layers. The nano-hardness of the intermetallic compounds could be controlled by adjusting the thickness of the Zn layer. Compared with the average nano-hardness of the AZ91D alloy, the Zn layer, the Al coating, and the intermetallic compounds (shown in Figure 9d) after the electrodeposition and heat treatment, the reinforcement in nano-hardness appeared very significant. The elastic modulus and the nano-hardness determined by the curves also increased with the Zn immersion time increases (Table 3), which proves that varying the thickness of the Zn layer can tune the nanomechanical properties of the Mg/Al intermetallic compounds.

In addition, after heat treatment, the variations of the elastic modulus and nano-hardness among the samples were more pronounced, which increased with increasing the Zn immersion time. Such an enhancement was due to the phase transformation and the strengthening effect of the β-phase after heat treatment, as abovementioned [26]. Generally, the second phases, β-phase and γ-phase, were believed to be harder than the Al coating and Zn film, and the β-phase was softer than the γ-phase [17,27]. The experiment results in this paper also give evidence to this conclusion. In the intermetallic compound layers, it can be assumed that the γ-phase and β-phase played equal roles in hardness. According to the volume percentage of these two phases, an average hardness could be calculated, which was approximately three times higher than that of the AZ91D substrate.

Overall, both the Zn layer and Al coating could enhance the nanomechanical properties of AZ91D. Also, the second phase formed by heat treatment can lead to further improvement. Admittedly, hardness is not a perfect measure of wear resistance, nor, in fact, is any single wear test since the wear of a given material may vary substantially depending on the mode of wear. However, in this work, since the requirement for a specific method of wear resistance is absent, hardness could also be taken as a generic measure of abrasion, scratch, and wear resistance. As a result, the increase of hardness implies that the mechanical and wear resistance of the Al coating was improved effectively.

### 3.4. Hydrogen Evolution Tests

**Figure 10:**
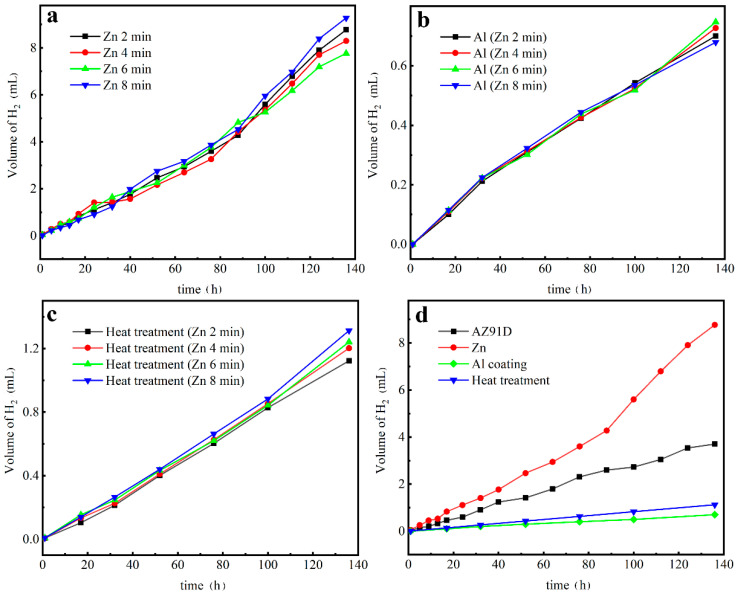
Hydrogen evolution curves for (**a**) Zn layers, (**b**) Al coatings, (**c**) Al coatings after heat treatment, and (**d**) the comparison of the curves of the AZ91D substrate, the Zn-immersed, the Al-coated, and the heat-treated samples.

Figure 10 shows the typical representation of the hydrogen evolution rate of the studied samples with increasing Zn immersion times in 3.5 wt.% NaCl solution. It can be seen from Figure 10a,b that as the Zn immersion time increased, there was no positive correlation between zinc dipping time and the amount and rate of hydrogen evolution. The results of the hydrogen evolution tests of the Zn layers and Al coatings exhibited no clear relationship with the Zn immersion times. A probable reason for this is that different corrosion protection mechanisms may have been involved in the Zn layers and Al coatings. After heat treatment, as shown in Figure 10c, the hydrogen evolution curves can be approximately considered as straight lines, which demonstrates that the corrosion of the intermetallic compounds shared the same mechanism. The amount of hydrogen generated by the corrosion of the Mg alloys increased with the increase in Zn immersion times, namely, with the increase of the ratio of the γ-phase and β-phase. It is indicated that the intermetallic compounds prepared by 2 min Zn immersion, which contained the most β-phase (Mg_17_Al_12_), were the least prone to corrosion in NaCl solutions.

For clarity, the comparison of the hydrogen evolution rates for the AZ91D substrate, the Zn-immersed, the Al-coated, and the heat-treated samples are shown in Figure 10d. The hydrogen evolution rate of the Zn layer sample was higher compared with that of the AZ91 substrate due to poor corrosion performance [28]. Meanwhile, the Al-coated sample presented the lowest rate, revealing that the Al coating provided the protection for the AZ91D substrate. In fact, the measurement of hydrogen evolution depends not on the dissolution of Al but that of Mg, since the cathodic reaction for Mg dissolution is responsible for hydrogen evolution [29]. Therefore, the dissolution of the substrate was suppressed by the Al coating. In addition, the hydrogen evolution rate of the heat-treated sample was slightly increased, indicating that the protection of the Al coating was undermined after heat treatment. The γ-phase that formed after heat treatment was believed to favor an oxygen reduction as the dominating cathodic reaction under the corrosion situation [19], and to be spontaneously passive in 3.5% NaCl solution [30,31]. However, the fact was that the hydrogen evolution rate slightly increased after heat treatment. There are two key factors that could be responsible for the phenomenon. One is that γ-phase can only provide limited protection during long-term corrosion since it is not a very inherent corrosion-resistant phase [19]. Another is that more Mg solutes were observed in the surface layer after heat treatment compared with the samples covered by electrodeposition Al coatings. Thus, it was inferred that the diffusion of Mg into the surface layer might lead to an increased rate, which can support water reduction as the cathodic reaction [32].

### 3.5. Potentiodynamic Testing

**Figure 11:**
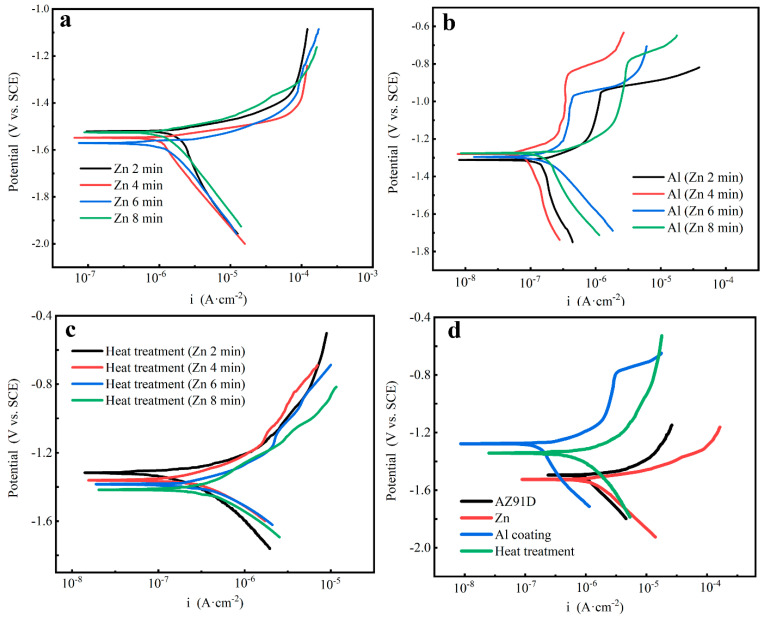
Potentiodynamic polarization curves of (**a**) the Zn layers, (**b**) the Al coatings, (**c**) the Al coatings after heat treatment, and (**d**) the comparison of the curves of the AZ91D substrate, the Zn-immersed, Al-coated, and the heat-treated samples.

Figure 11 shows the potentiodynamic polarization curves of the different studied samples; other detail parameters calculated by the Tafel extrapolation are summarized and shown in Table 4. Similarly, it can be seen that the corrosion current density (i_corr_) and corrosion potential (E_corr_) at the OCP of the Zn layers and the Al coatings did not appear to be regular changes as the Zn immersion times increased. In contrast, the i_corr_ of the Mg/Al intermetallic compounds increased from 42.19 μA·cm^−2^ to 60.57 μA·cm^−2^, and the E_corr_ shifted towards the negative direction from −1.35 V to −1.41 V as the Zn immersion times increased from 2 min to 8 min. The variations of the content of the different intermetallic compounds β-phase (Mg_17_Al_12_) and γ-phase (Mg_2_Al_3_), which were attributed to the control of the Zn immersion layer thickness, contributed to the difference in corrosion resistance. As the Zn immersion time increased, the Zn layers got thicker and the composition of the β-phase (Mg_17_Al_12_) decreased but that of the γ-phase (Mg_2_Al_3_) increased; as a result, the corrosion resistance declined gradually. The experimental results of the potentiodynamic testing were in accordance with those of the hydrogen evolution tests.

The typical samples after each process were selected, and the comparison of potentiodynamic polarization curves of different samples is shown in Figure 11d. The i_corr_ and the E_corr_ at the OCP of AZ91D were 103.81 μA·cm^−2^ and −1.51 V, respectively. As the Zn layer was immersed in the Mg alloy matrix, the i_corr_ increased and the E_corr_ became more negative, indicating that the Zn layer could not protect the Mg alloy matrix. After the electrodeposition of Al coatings, the samples showed a polarization response of pure Al, and remarkable passivation ranges were observed in the potentiodynamic polarization curves. A much lower free corrosion current density than the Mg alloy substrate confirmed that the electrodeposition Al coatings could improve corrosion resistance effectively. After heat treatment, the passivation behavior disappeared expectantly since the electrodeposition Al coatings were replaced by the intermetallic compounds β-phase (Mg_17_Al_12_) and γ-phase (Mg_2_Al_3_). The free corrosion current density increased slightly and a little negative shift occurred in the free corrosion potential compared with the electrodeposition Al coatings, demonstrating that there was little difference before and after heat treatment in corrosion resistance. However, the free corrosion current density of the heat-treated sample was still much smaller than that of the Mg alloy substrate. The experiment results of the potentiodynamic testing are consistent with the trend shown in the hydrogen evolution tests (Figure 10) and suggest that the corrosion resistance of the heat-treated samples was much better than that of the Mg alloy.

### 3.6. Comprehensive Assessment

**Figure 12:**
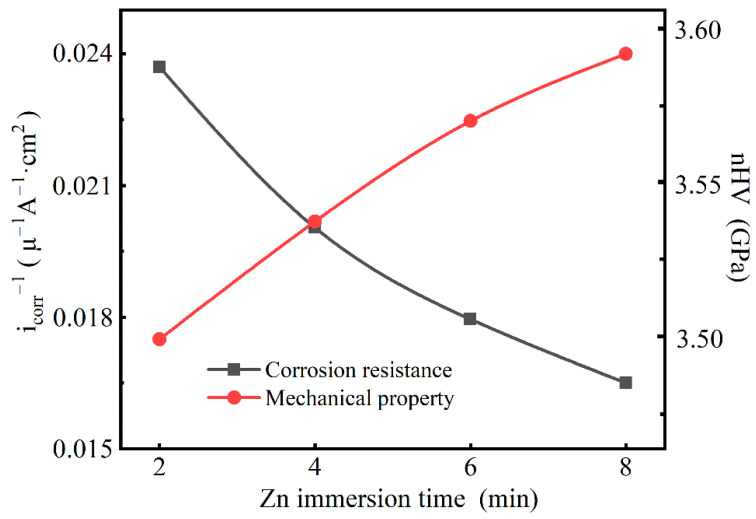
The qualitative mechanical property and corrosion resistance variation of the Mg/Al intermetallic compounds β-phase (Mg_17_Al_12_) and γ-phase (Mg_2_Al_3_), with various Zn immersion times (I) Zn 2 min, (II) Zn 4 min, (III) Zn 6 min, and (IV) Zn 8 min.

According to the previous experimental results, the relationship between the thickness of the Zn layer and the mechanical performance of the obtained intermetallic compounds was established and is presented in Figure 12. The average nano-hardness value of the intermetallic compounds and the reciprocal value of the free corrosion current density were selected as the qualitative criteria of the mechanical properties and corrosion resistance. Figure 12 reflects the inter-relationship between the thickness of the Zn layer and the mechanical property and corrosion resistance of the Mg/Al intermetallic compounds, qualitatively.

It can be seen that with the increases in the Zn immersion times, the mechanical properties were gradually improved, while their corrosion resistance got worse. In practical applications, the requirements for material properties are multiple. In order to evaluate the properties of the material comprehensively and completely, it is necessary to balance the relationship between the mechanical properties and corrosion resistance. A preliminary and comprehensive assessment can be made for the comprehensive evaluation of the mechanical properties and corrosion resistance of a material. When the time of Zn immersion was too short (2 min) or too long (8 min), poor corrosion resistance or mechanical properties of the Mg/Al intermetallic compounds β-phase (Mg_17_Al_12_) and γ-phase (Mg_2_Al_3_) were serious shortcomings that hindered further application of the materials. In contrast, when the Zn immersion time was 4 min, the obtained intermetallic compounds had relatively optimal comprehensive properties. Moreover, controlling the thickness of the Zn immersion layer to adjust the different components of the intermetallic compounds could be the simplest method to achieve the optimal comprehensive performance, which may be of practical importance for surface modification of the materials.

## 4. Conclusions

Electrodeposition of Al coatings on AZ91 alloys from AlCl_3_–NaCl–KCl molten salts was achieved to prevent the substrate from rapidly corroding. Post-plating heat treatment processes were explored to improve coating adhesion and to maintain corrosion resistance. The conclusions can be drawn as follows:

(1) Different thicknesses of Zn films immersed on AZ91 alloy that varied from 0.2 μm to 1.8 μm were achieved by controlling the immersion times from 2 min to 8 min. The compact and uniform Al coating with a thickness of 7 ± 3 μm was electrodeposited on the Zn film, which exhibited no clear relationship with the thickness of the Zn layer. After heat treatment at 350 °C, the Al coating and the Zn layer were transformed to Mg/Al intermetallic compounds β-phase (Mg_17_Al_12_) and γ-phase (Mg_2_Al_3_).
(2) Evaluated by nanoindentation testing, the nano-hardness properties of the AZ91D increased from 1.1 ± 0.1 GPa to 1.5 ± 1.0 GPa by Zn immersion, and to 2.4 ± 0.5 GPa by the electrodeposition of Al coatings. In addition, the obtained Mg/Al intermetallic compounds induced by heat treatment led to a further improvement to reach 3.5 ± 0.1 GPa.
(3) The hydrogen evolution tests and the potentiodynamic polarization proved that the Zn layer could not protect the Mg alloy matrix, while the electrodeposition Al coatings and heat treatment could improve corrosion resistance effectively. Compared with the Al coatings, the corrosion resistance of the Mg/Al intermetallic compounds β-phase (Mg_17_Al_12_) and γ-phase (Mg_2_Al_3_) was a little bit worse.
(4) With the increase of the Zn immersion times, the thickness of the Zn layer increased and the mechanical properties were gradually improved, while their corrosion resistance got worse. When the Zn immersion time was 4 min, the obtained intermetallic compounds had relatively optimal comprehensive properties.

## Figures and Tables

**Table 1:** The solution compositions and operation conditions of Zn immersion.

Process	Bath Compositions	Content	Conditions	Time
Pickling	C_6_H_8_O_7_	40 g/L	Room Temperature	2 min
Conditioning	NaOH	200 g/L	Room Temperature	10 min
Activation	C_6_H_8_O_7_	20 g/L	Room Temperature	40 s
Zn immersion	ZnSO_4_·7H_2_O	30 g/L	80 °C	2~8 min
	Na_4_P_2_O_7_·10H_2_O	120 g/L		
	NaF	5.5 g/L		
	Na_2_CO_3_	5.5 g/L		

**Table 2:** The EDXS of the Al coating after 350 °C heat treatment for 4 h.

Point	Al wt. %	Zn wt. %	Mg wt. %	Al at. %	Zn at. %	Mg at. %
1	62.1	0.0	37.9	59.3	0.0	40.7
2	61.9	0.0	38.1	59.1	0.0	40.9
3	61.3	0.0	38.8	58.4	0.0	41.6
4	61.1	0.0	38.9	58.3	0.0	41.7
5	39.8	0.9	59.3	41.4	2.8	55.8
6	36.9	1.5	61.6	38.5	3.7	57.8
7	0.0	0.0	100.0	0.0	0.0	100.0
8	0.0	0.0	100.0	0.0	0.0	100.0

**Table 3:** The nanomechanical properties of the AZ91D substrate, the Zn film, the Al coating, β-phase (Mg_17_Al_12_) and γ-phase (Mg_2_Al_3_).

Unit	AZ91D	–	Zn	Al	β-Phase (Mg_17_Al_12_)	γ-Phase (Mg_2_Al_3_)
nHV (GPa)	1.12 ± 0.09	Zn 2 min	1.40 ± 0.04	2.35 ± 0.06	3.51 ± 0.05	3.49 ± 0.10
		Zn 4 min	1.45 ± 0.02	2.40 ± 0.08	3.55 ± 0.04	3.52 ± 0.08
		Zn 6 min	1.53 ± 0.05	2.41 ± 0.07	3.56 ± 0.04	3.57 ± 0.07
		Zn 8 min	1.57 ± 0.03	2.44 ± 0.06	3.58 ± 0.06	3.59 ± 0.08
EIT (GPa)	40.20 ± 8.40	Zn 2 min	49.72 ± 0.03	66.73 ± 0.05	65.91 ± 0.08	63.15 ± 0.06
		Zn 4 min	51.54 ± 0.04	67.51 ± 0.06	66.32 ± 0.07	63.89 ± 0.05
		Zn 6 min	52.71 ± 0.04	67.76 ± 0.05	66.67 ± 0.06	64.28 ± 0.08
		Zn 8 min	55.31 ± 0.02	68.25 ± 0.07	66.78 ± 0.06	64.81 ± 0.06

**Table 4:** Corrosion characteristics obtained by polarization in 3.5% NaCl solution of the AZ91D, the Zn layers, the Al coatings, and the Al coatings after heat treatment.

**Unit**	**AZ91D**	**Zn layer** **Zn 2 min**	**Zn layer** **Zn 4 min**	**Zn layer** **Zn 6 min**	**Zn layer** **Zn 8 min**
E_corr_ (V)	−1.51	−1.52	−1.55	−1.56	−1.52
i_corr_ (μA·cm^−2^)	103.81	125.18	118.07	120.90	122.52
**Unit**		**Al-coated** **Zn 2 min**	**Al-coated** **Zn 4 min**	**Al-coated** **Zn 6 min**	**Al-coated** **Zn 8 min**
E_corr_ (V)		−1.31	−1.27	−1.29	−1.27
i_corr_ (μA·cm^−2^)		35.79	33.44	41.38	37.49
**Unit**		**Heat Treatment** **Zn 2 min**	**Heat Treatment** **Zn 4 min**	**Heat Treatment** **Zn 6 min**	**Heat Treatment** **Zn 8 min**
E_corr_ (V)		−1.35	−1.37	−1.39	−1.41
i_corr_ (μA·cm^−2^)		42.19	49.90	55.67	60.57

## Data Availability

The data presented in this study are available in this article.
